# Cathepsin E Deficiency Impairs Autophagic Proteolysis in Macrophages

**DOI:** 10.1371/journal.pone.0082415

**Published:** 2013-12-05

**Authors:** Takayuki Tsukuba, Michiyo Yanagawa, Tomoko Kadowaki, Ryosuke Takii, Yoshiko Okamoto, Eiko Sakai, Kuniaki Okamoto, Kenji Yamamoto

**Affiliations:** 1 Division of Oral Pathopharmacology, Graduate School of Biomedical Sciences, Nagasaki University, Nagasaki, Japan; 2 Department of Oral Surgery, Graduate School of Medicine, Yamaguchi University, Ube, Yamaguchi, Japan; 3 Department of Biochemistry and Molecular Biology, Graduate School of Medicine, Yamaguchi University, Ube, Yamaguchi, Japan; 4 Department of Biochemistry, Daiichi University College of Pharmaceutical Sciences, Fukuoka, Japan; 5 Proteolysis Research Laboratory, Graduate School of Pharmaceutical Sciences, Kyushu University, Fukuoka, Japan; The Hospital for Sick Children and The University of Toronto, Canada

## Abstract

Cathepsin E is an endosomal aspartic proteinase that is predominantly expressed in immune-related cells. Recently, we showed that macrophages derived from cathepsin E-deficient (*CatE* ^*−/−*^) mice display accumulation of lysosomal membrane proteins and abnormal membrane trafficking. In this study, we demonstrated that *CatE* ^*−/−*^ macrophages exhibit abnormalities in autophagy, a bulk degradation system for aggregated proteins and damaged organelles. *CatE* ^*−/−*^ macrophages showed increased accumulation of autophagy marker proteins such as LC3 and p62, and polyubiquitinated proteins. Cathepsin E deficiency also altered autophagy-related signaling pathways such as those mediated by the mammalian target of rapamycin (mTOR), Akt, and extracellular signal-related kinase (ERK). Furthermore, immunofluorescence microscopy analyses showed that LC3-positive vesicles were merged with acidic compartments in wild-type macrophages, but not in *CatE* ^*−/−*^ macrophages, indicating inhibition of fusion of autophagosome with lysosomes in *CatE* ^*−/−*^ cells. Delayed degradation of LC3 protein was also observed under starvation-induced conditions. Since the autophagy system is involved in the degradation of damaged mitochondria, we examined the accumulation of damaged mitochondria in *CatE* ^*−/−*^ macrophages. Several mitochondrial abnormalities such as decreased intracellular ATP levels, depolarized mitochondrial membrane potential, and decreased mitochondrial oxygen consumption were observed. Such mitochondrial dysfunction likely led to the accompanying oxidative stress. In fact, *CatE* ^*−/−*^ macrophages showed increased reactive oxygen species (ROS) production and up-regulation of oxidized peroxiredoxin-6, but decreased antioxidant glutathione. These results indicate that cathepsin E deficiency causes autophagy impairment concomitantly with increased aberrant mitochondria as well as increased oxidative stress.

## Introduction

Cathepsin E is an intracellular aspartic proteinase that is predominantly distributed in immune-related cells [1–3]. The subcellular localization of cathepsin E is mainly in endosomal compartments in antigen-presenting cells such as macrophages, dendritic cells, and microglia [4–7]. Our previous study has demonstrated that cathepsin E-deficient (*CatE* ^*−/−*^) mice spontaneously developed atopic dermatitis-like skin lesions when reared under conventional conditions, but they did not develop these lesions under specific pathogen-free conditions [8]. This finding suggests that this phenotype was triggered by some environmental factor(s), including pathogenic microorganisms. Indeed, we recently reported that *CatE* ^*−/−*^ mice exhibited increased susceptibility to bacterial infection with *Staphylococcus aureus* or *Porphyromonas gingivalis* [9]. However, the precise molecular mechanisms inducing aberrant immune responses in *CatE* ^*−/−*^ mice remain unclear.

Recently, we reported that peritoneal macrophages from *CatE* ^*−/−*^ mice display a novel type of lysosomal storage disorder characterized by intracellular accumulation of major lysosomal membrane sialoglycoproteins (LAMP-1 and LAMP-2) [10]. In *CatE* ^*−/−*^ macrophages, the lysosomal membrane glycoprotein accumulation induced a concomitant elevated lysosomal pH and enhanced extracellular secretion of various lysosomal hydrolases [10,11]. The accumulated membrane proteins in *CatE* ^*−/−*^ macrophages seem to induce secondary abnormalities in membrane trafficking. In fact, the cell surface levels of Toll-like receptors, chemotactic receptors, and adhesion receptors were significantly lower in *CatE* ^*−/−*^ macrophages compared with those in wild-type cells [12]. Therefore, we hypothesized that the abnormal membrane trafficking in *CatE* ^*−/−*^ macrophages influences both extracellular and intracellular events such as cell surface receptor expression and autophagy, respectively.

Autophagy (macro-autophagy) is a process by which the dynamic rearrangement of cellular membranes allows portions of the cytoplasm to be delivered to the lysosomes, where they are degraded [13]. Recent studies have revealed that autophagy is necessary for certain aspects of cellular homeostasis, such as prevention of neurodegeneration [14–16], and for various host defense mechanisms including prevention of tumor progress [17], clearance of pathogenic bacteria and viruses [18], and antigen presentation [19]. Therefore, autophagy is essential for the maintenance of the fundamental cell functions in immune-related cells. Given that a lack of cathepsin E induces various abnormal membrane trafficking events in immune-related cells, it is possible that cathepsin E deficiency causes impairment of autophagy concomitant with the accumulation of toxic proteins or damaged organelles. In this study, we demonstrated that cathepsin E deficiency causes an impaired autophagic flux and an increase in the number of aberrant mitochondria in macrophages.

## Materials and Methods

Antibodies to mouse LC3 and beclin-1 were purchased from Medical & Biology Laboratories (Nagoya, Japan). The antibody to p62/SQSTM1 was purchased from Progen Biotechnique (Heidelberg, Germany). The antibody to ubiquitin-protein conjugates was obtained from Biomol International, PA, USA. Antibodies to mammalian target of rapamycin (mTOR), p-mTOR(Ser2448), Akt, p-Akt(Ser473), extracellular signal-related kinase (ERK), and p-ERK (Tyr204, Ser473), S6K, p-S6K (Thr 389), adenosine monophosphate kinase (AMPK)α, p-AMPKα (Thr172), were purchased from Cell Signaling Technology (Beverly, MA, USA). Antibodies to actin were from Santa Cruz Biotechnology (Santa Cruz, CA, USA). The ATP assay kit (CellTiter-Gio Luminescent cell viability assay) was purchased from Promega (Madison, WI, USA). Cell counting kit-8 (CCK-8) was from Dojindo (Kumamoto, Japan). Baf and 3-MA were purchased from Wako Pure Chemicals (Tokyo, Japan). LysoTracker Red DND-99 and Alexa Fluor 488 goat anti-rabbit IgG were purchased from Molecular Probes/Invitrogen (Eugene, Oregon, USA). FCCP was from Sigma-Aldrich Inc (Tokyo, Japan).

### Animals

Wild-type and *CatE* ^*−/−*^ mice with the C57BL/6 genetic background were used as described previously [10]. All animals were maintained according to the guidelines of the Japanese Pharmacological Society. The animals and all experiments were approved by the Animal Research Committees of the Graduate School of Biomedical Sciences, Nagasaki University (Permit Number: 1205080984-2). All surgical procedures were performed under ether anesthesia, and every effort was made to minimize suffering.

### Preparation of peritoneal macrophages

Thioglycolate-elicited peritoneal macrophages were isolated from mice as described previously [10]. Briefly, 8- to 14-week-old mice were peritoneally injected with 4.05% thioglycolate (2 mL/mouse). Three and a half days later, peritoneal exudate cells were isolated from the peritoneal cavity by washing with phosphate-buffered saline (PBS). The cells were incubated in RPMI 1640 medium supplemented with 10% fetal bovine serum (FBS), penicillin (50 units/mL), and streptomycin (50 µg/mL) at 37°C with 5% CO_2_. After incubation for 2 h, non-adherent cells were removed by washing 3 times with Ca^2+^/Mg^2+^-free PBS. Peritoneal macrophages isolated as adherent MAC-2-positive cells were obtained at a purity of greater than 95%.

### Preparation of cell lysates

For the preparation of cell lysates, the cells were washed twice with PBS, removed from the plates with a rubber scraper, and centrifuged at 300 ×*g* for 5 min. The precipitated cells were resuspended in PBS containing 0.1% Triton X-100, and then subjected to sonication for 1 min at 4°C followed by centrifugation at 100,000 ×*g* for 1 h. The supernatant fraction was referred to as the cell lysate. For sodium dodecyl sulfate polyacrylamide gel electrophoresis (SDS-PAGE) or immunoblot analyses, the supernatant was precipitated with TCA at a final concentration of 5% and centrifuged at 12,000 ×g for 15 min after incubation on ice for 15 min. After washing with ice-cold acetone and evaporating with air, the pellets were suspended in the buffer for SDS-PAGE. The cells were washed twice with PBS, removed from the plates by pipetting, and then subjected to centrifugation at 300 × *g* for 5 min. The precipitated cells were suspended in PBS containing 0.05% Triton X-100, sonicated 3 times for 5 s at 4°C, and subjected to centrifugation at 120,000 × *g* for 30 min at 4°C. The supernatant was used as the cell lysate. 

### Gel electrophoresis and western blot analysis

SDS-PAGE and western blot were performed as described previously [20]. The quantification of the immunoreactive bands was analyzed by LAS-1000 and Image Gauge software (Fuji Photo Film Co., Ltd., Tokyo, Japan)

### Bulk protein degradation assay

The bulk protein degradation assay was performed according to the method described by Fujita et al. [21] with some modifications. Briefly, cells (1 × 10^6^ cells) were seeded in 35 mm culture dishes and incubated overnight. The medium were substituted with labeling medium containing [^14^C] leucine (0.5 Ci/mL per dish) for 24 h in 2.0 mL of RPMI 1640 medium supplemented with 10% FBS. Cells were then incubated in 2.0 mL of chase medium (DMEM supplemented with 10% FBS and 10 mM unlabeled leucine) and further incubated for 4 h to remove the contribution of short-lived proteins. After a 4-h incubation, the cells were lysed in 1 mL of 10 mM Tris-HCl buffer at pH 7.5 containing 150 mM NaCl, 5 mM EDTA, 1% Triton X-100, 0.5% sodium deoxycholate, and 0.02% sodium azide. The cell lysates and the collected media were mixed with trichloroacetic acid (TCA) to a final TCA concentration of 10%. After centrifugation at 12,000 ×*g* for 5 min, TCA-insoluble cell lysates were dissolved in 1 mL of 1 N NaOH. The radioactivity of the TCA-insoluble cell lysates and the TCA-soluble media was determined by a scintillation counter. The rate of degradation of the bulk long-lived protein was calculated as the total activity of the media divided by that of the cell lysates.

### Immunofluorescence microscopy

The cells were grown on glass cover slips and then preincubated with LysoTracker-Red DND-99 for 30 min. After washing with PBS, the cells were fixed with 4.0% paraformaldehyde in PBS for 30 min at room temperature. The fixed cells were then washed with 50 mM NH_4_Cl in PBS for quenching, and permeabilized with 0.3% Tween-20 in PBS for 10 min. The cells were incubated with 1% bovine serum albumin and 1% normal goat serum in PBS for 3 h and subsequently incubated with anti-LC3 antibody overnight at 4°C, followed by Alexa Fluor 488 goat anti-rabbit IgG as a secondary antibody. The samples were inspected by microscopy using a laser-scanning confocal imaging system (LSM510 META; Carl Zeiss, Co., Ltd).

### Determination of mitochondrial membrane potential in macrophage*s*

The mitochondrial membrane potential was measured using 5,5′,6,6′-tetrachloro-1,1′,3,3′ tetraethylbenzimidazolylcarbocyanine iodide (JC-1; Molecular Probes/Invitrogen) according to a published protocol with some modifications. The peritoneal macrophage suspension (2 × 10^5^ cells/100 μL) was incubated on ice for 15 min with 10 μg/mL of JC-1 in PBS containing 2.5% FBS and 0.01% NaN_3_ (buffer A). After washing with buffer 3 times, flow cytometry analyses were performed using an Epics XL flow cytometer (Beckman Coulter, Brea, CA, USA). 

### Isolation of mitochondria

Macrophages grown on 100-mm plates were washed twice with the fraction buffer (250 mM sucrose, 5 mM HEPES, 50 mM KCl, 6 mM MgCl_2_, 1 mM EDTA, pH 7.5) and scraped with soft rubber scrapers. The cells were then homogenized with 3 strokes in a glass Dounce homogenizer. The homogenates were centrifuged at 1,000 ×*g* for 10 min at 4°C, and the resultant supernatants were centrifuged at 10,000 ×*g* for 10 min at 4°C, and used as crude mitochondrial preparations.

### Two-dimensional electrophoresis and proteomic analyses

Two-dimensional (2-D) electrophoresis and proteomic analyses were performed as described previously (10). Briefly, the cell lysates from macrophages were applied to immobilized pH gradient (IPG) strips (Amersham Biosciences, Piscataway, NJ, USA) and then subjected to isoelectric focusing using a Multiphor II (Amersham Pharmacia Biotech). After isoelectric focusing, the strips were equilibrated for 15 min in 50 mM Tris-HCl, pH 8.8, containing 6 M urea, 30% glycerol, 1% SDS, and 64 mM dithiothreitol and subsequently immersed for 15 min in the same buffer containing 135 mM iodoacetamide instead of dithiothreitol. The strips were then transferred onto 10% SDS-polyacrylamide gels. After electrophoresis, the gels were silver-stained for proteins. Peptide mass mapping was performed by recording the peptide mass fingerprints of typical in-gel digests of the corresponding gel bands by using matrix-assisted laser desorption ionization time-of-flight mass spectrometer (MS; AXIMA-CFR plus, Shimadzu, Tsukuba, Japan) and the subsequent use of the Mascot search engine (Matrix Science, Tokyo, Japan).

### Measurement of oxidative burst by macrophages

Oxidative burst production was measured according to the method described previously with some modification [22]. Briefly, the macrophage cell suspension (1×10^7^ cells /mL) was preincubated at 37°C. Zymosan A suspended in PBS (20 mg/mL) was boiled for 10 min and washed. The zymosan suspension was incubated with an equal volume of mouse serum at 37°C for 30 min. The particles were washed twice with PBS and suspended in their original volume in PBS. The cuvette for the reaction mixture, which contained 0.1 mL of freshly diluted luminol solution (0.2 mM), 0.1ml of the macrophage suspension (2×10^7^ cells/mL) and 0.1 mL of the opsonized zymosan (20 mg/mL) was maintained at 37° C in the Luminophotometer TD-4000 (LABO Science Co., Tokyo, Japan). The intensity of light emitted in the cuvette was recorded automatically. The chemiluminescent response corresponded to the peak of the curve.

### Measurement of hydrogen peroxide production (H_2_O_2_) by macrophages

Determination of H_2_O_2_ production in the culture media by macrophages was performed using an Amplex Redassay kit (Molecular Probes). 

### Measurement of reduced glutathione (GSH)

GSH was spectrophotometrically determined with nicotinamide adenine dinucleotide phosphate (NADPH) at 412 nm by measuring the intensity of color development with DTNB according to the method of described by Beutler et al. [23] with some modification. 

### Statistical analysis

Quantitative data are presented as mean + standard deviation (SD). The statistical significance of differences among mean values was assessed by Student’s *t*-test. *P* values of <0.05 were considered statistically significant.

## Results

### Cathepsin E deficiency increases autophagy marker proteins in macrophages

**Figure 1:**
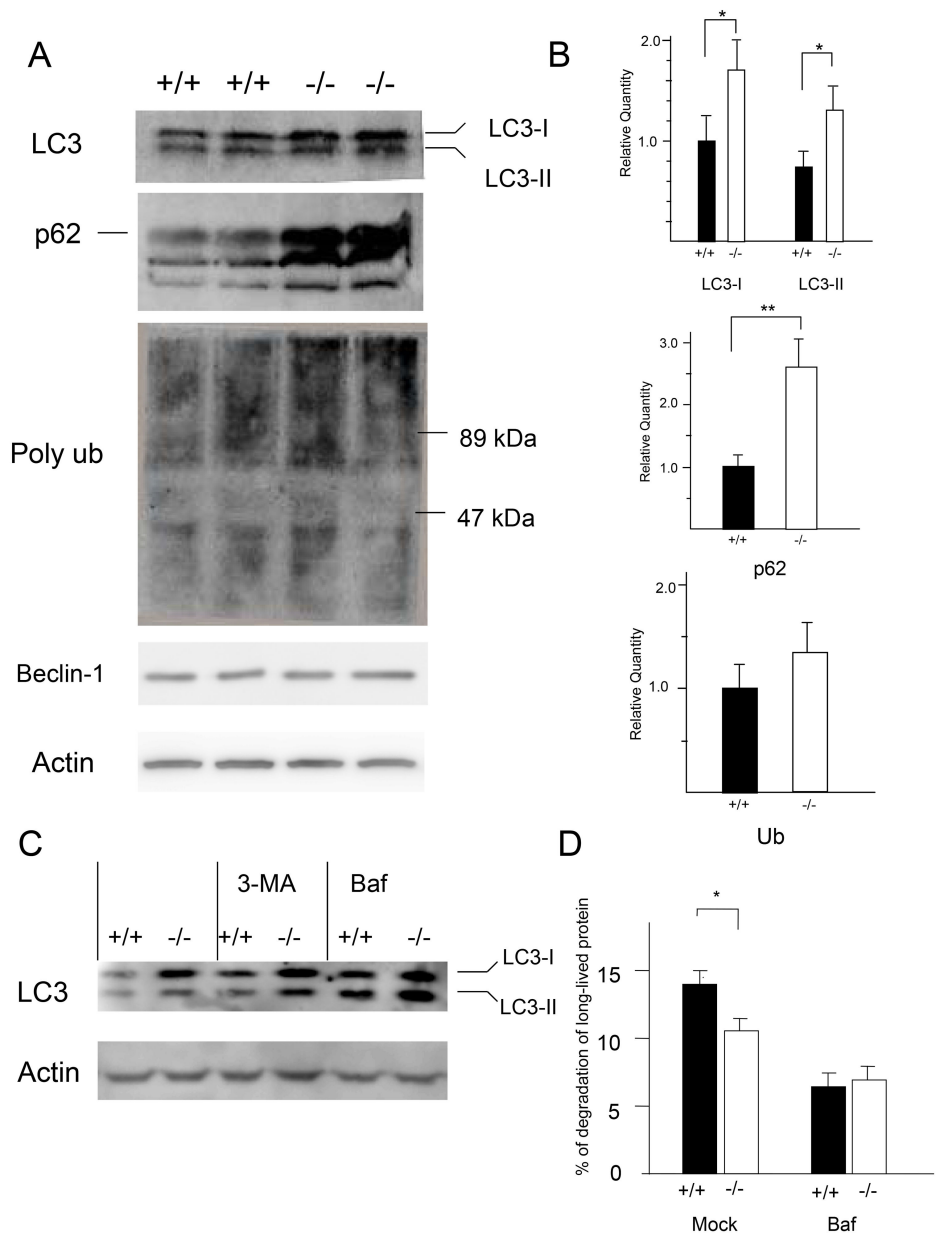
Levels of autophagy-related proteins in wild-type and CatE^−/−^ macrophages. (**A**) After a 1-day culture, the cell lysates (100 µg protein for each) derived from wild-type (+/+) and CatE^−/−^ macrophages (−/−) were subjected to SDS-PAGE followed by western blotting with specific antibodies to LC3, p62, ubiquitin, and actin. The data indicate the representative western blotting of five independent experiments. (**B**) Densitometric analysis for the quantification of each protein in the cell lysate of both cell types. The arbitrary density unit was defined as the relative chemiluminescence intensity per mm^2^ measured by LAS1000. The data are indicated as the mean ± SD values from 5 independent experiments. *P < 0.05 for the indicated comparisons. (**C**) The cells were cultured for 2 days, and then treated with the lysosomal inhibitors Baf (0.5 μM) and 3-MA (5 mM) or without for 12 h. After lysis, samples were subjected to SDS-PAGE followed by western blotting with antibodies to LC3 or actin. The data indicate the representative western blotting of 4 independent experiments. (**D**) Pulse-chase experiments using [^14^C] leucine were performed to study bulk long-lived proteins. The radioactivity of the TCA-insoluble cell lysates and TCA-soluble media was determined, and was used to calculate as the rate of degradation of the long-lived proteins. The data are shown as the percent of degradation of long-lived proteins from 3 independent experiments. *P < 0.05 for the indicated comparisons.

To determine whether cathepsin E is involved in autophagy in macrophages, we performed western blot analysis of microtubule-associated protein 1A/1B light chain 3 (LC3), an autophagy marker protein [24]. LC3 is detected as 2 bands by western blotting; one band represents LC3-I, a cytosolic form of approximately 16 kDa, the other band consists of LC3-II, which is conjugated to phosphatidylethanolamine, as a membrane-associated form of approximately 14 kDa [25]. Western blot analysis revealed higher LC3-I and LC3-II protein levels in *CatE* ^*−/−*^ macrophage lysates than in wild-type macrophage lysates (Figure 1A). Densitometric analysis for the quantification of protein levels showed that LC3-I and LC3-II protein levels were significantly higher in *CatE* ^*−/−*^ macrophages than in wild-type cells (Figure 1B). 

Besides LC3, p62/SQSTM1 is also used as an autophagy marker, because p62 associates directly with LC3 to promote degradation of the ubiquitinated protein aggregates by autophagy [26]. Consequently, blocking autophagy leads to an increase in protein levels and aggregation of p62 [27,28]. The p62 protein levels in *CatE* ^*−/−*^ macrophages were markedly higher than those in wild-type cells (Figure 1A and B).

Furthermore, we investigated the levels of polyubiquitinated proteins in *CatE* ^*−/−*^ macrophages, because it has been reported that blockage of autophagy induces cellular polyubiquitinated protein accumulation [27,28]. The high-molecular-weight (>50 kD) polyubiquitinated protein levels of *CatE* ^*−/−*^ macrophages were slightly higher than those of wild-type cells, although the difference was not statistically significant (Figure 1 A and B). Thus, the increased levels of autophagy-related proteins LC3 and p62, and polyubiquitinated proteins in *CatE* ^*−/−*^ macrophages, suggest that cathepsin E deficiency induces abnormalities in autophagy.

Using a different approach, to further confirm whether cathepsin E deficiency inhibits autophagy, we treated macrophages with lysosomotropic agents such as bafilomycin A_1_ (Baf) and 3-methyladenine (3-MA). Baf is a vacuole-type ATPase inhibitor, whereas 3-MA is a class III phosphatidylinostol 3-kinase inhibitor. Treatment of wild-type and *CatE* ^*−/−*^ macrophages with Baf or 3-MA induced the accumulation of LC3-I and LC3-II, respectively (Figure 1C). Specifically, the patterns of 3-MA treated wild-type cells mimicked those of *CatE* ^*−/−*^ cells without the inhibitor (Figure 1C). The Baf treatment significantly increased LC3-II levels in both wild-type cells and *CatE* ^*−/−*^ cells. These data suggest that cathepsin E deficiency inhibits autophagy (Figure 1C).

### CatE^−/−^ macrophages macrophages accumulate long-lived proteins

To further examine the differences between wild-type and *CatE* ^*−/−*^ macrophages, we performed pulse-chase experiments with [^14^C] leucine to assess the turnover of long-lived proteins in both cell types with and without Baf treatment (Figure 1D). We compared the radioactivity of the trichloroacetic acid (TCA)-insoluble cell lysate and TCA-soluble medium fractions. The percentage of radio-labeled proteins that were released into the media of *CatE* ^*−/−*^ macrophages was significantly lower than that of wild-type cells (Figure 1D). Under the conditions used in our study, Baf treatment decreased the release of TCA-soluble radioactivity in both wild-type and *CatE* ^*−/−*^ macrophages (Figure 1D). These results also suggest that *CatE* ^*−/−*^ macrophages possess relatively lower autophagic degradation activity.

### Cathepsin E deficiency alters autophagy-related signaling

**Figure 2:**
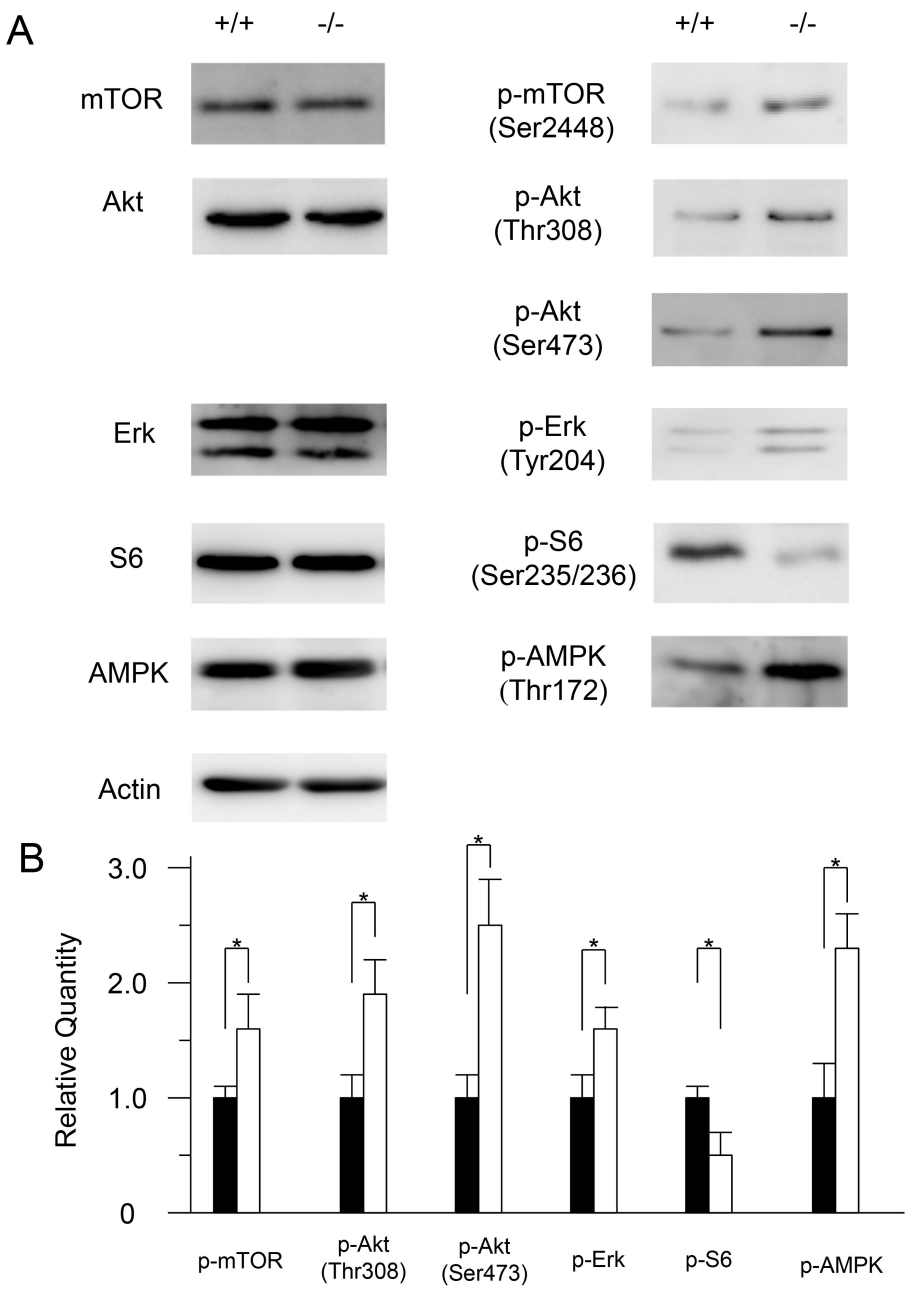
Comparison of Akt/mTOR and ERK signaling pathways in wild-type and *CatE* ^*−/−*^ macrophages. (**A**) The cell lysates (100 µg protein for each) derived from wild-type (+/+) and *CatE* ^*−/−*^ macrophages (−/−) were subjected to SDS-PAGE followed by western blotting with specific antibodies to mTOR, p-mTOR, Akt, p-Akt(Thr308), p-Akt(Ser473), ERK, p-ERK, S-6, p-S6, AMPK, p-AMPK and actin. The data indicate the representative western blotting of 3 independent experiments. (**B**) Densitometric analysis for the quantification of each protein in the cell lysate of both cell types. The arbitrary density unit was defined as the relative chemiluminescence intensity per mm^2^ measured by LAS1000. The data are indicated as the mean ± SD values from 3 independent experiments. *P < 0.05 for the indicated comparisons.

It has been reported that autophagy is regulated by various signaling pathways such as those involving or dependent on mTOR, its substrate p70S6 kinase, Akt, ERK, AMPK [29,30]. Therefore, we analyzed these signaling molecules by western blotting. The protein levels of mTOR, Akt, ERK, and AMPK of both wild-type and *CatE* ^*−/−*^ macrophages were similar (Figure 2). However, the phosphorylation levels of mTOR, Akt, ERK and AMPK were apparently increased in *CatE* ^*−/−*^ macrophages compared to wild-type cells (Figure 2). By contrast, the phosphorylation of S6 was decreased in *CatE* ^*−/−*^ macrophages (Figure 2). These results suggest that the autophagy-related signaling was perturbed in *CatE* ^*−/−*^ macrophages.

### Cathepsin E deficiency impairs fusion of autophagosomes with lysosomes

**Figure 3:**
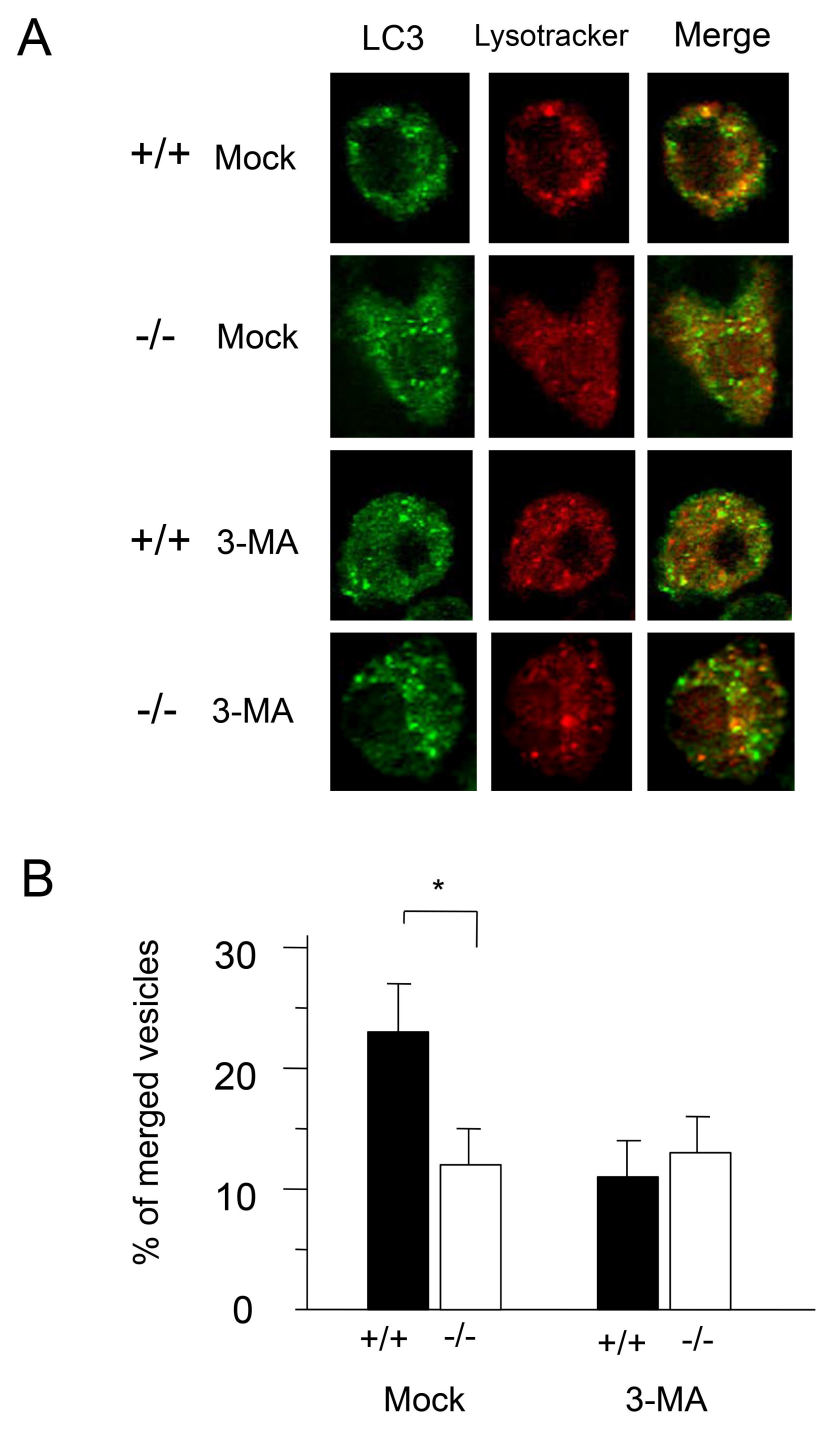
Immunofluorescence microscopy of LC3 and LysoTracker in wild-type and *CatE* ^*-/-*^macrophages. (**A**) Cells on glass cover-slips were preincubated with LysoTracker-Red for 30 min, subsequently fixed, permeabilized with 0.3% Tween-20 in PBS, and allowed to react with anti-LC3 antibody. The cells were then incubated with a fluorescence-labeled secondary antibody and visualized by confocal laser microscopy. (**B**) Based on the data from immunofluorescence microscopy, the number of LC3- or LysoTracker-positive vesicles per cell was counted. The data are shown as percent merged vesicles per cell and acquired from 3 independent experiments. *P < 0.05 for the indicated comparisons.

To compare the membrane fusion of autophagosomes with lysosomes in *CatE* ^*−/−*^ and wild-type macrophages, we analyzed the localization of LC3 and LysoTracker, a fluorescent probe for acidic compartments, such as lysosomes and endosomes by confocal fluorescence microscopy (Figure 3). Upon mock treatment, LC3-positive vesicles in wild-type macrophages were merged with LysoTracker-positive vesicles, whereas those in *CatE* ^*−/−*^ macrophages did not colocalize with LysoTracker-positive vesicles (Figure 3A). However, when both types of cells were treated with 3-MA, the number of merged vesicles in wild-type cells were apparently decreased, while those in *CatE* ^*−/−*^ cells remained unaffected (Figure 3A). When the number of LC3-positive vesicles per cell was counted upon mock treatment, the number of those that merged with LysoTracker-positive vesicles was significantly higher in wild-type cells than in *CatE* ^*−/−*^ cells (Figure 3B). These morphological results also indicate that cathepsin E deficiency inhibits the transport of LC3 to acidic compartments, implying inhibition of autophagy in *CatE* ^*−/−*^ cells.

**Figure 4:**
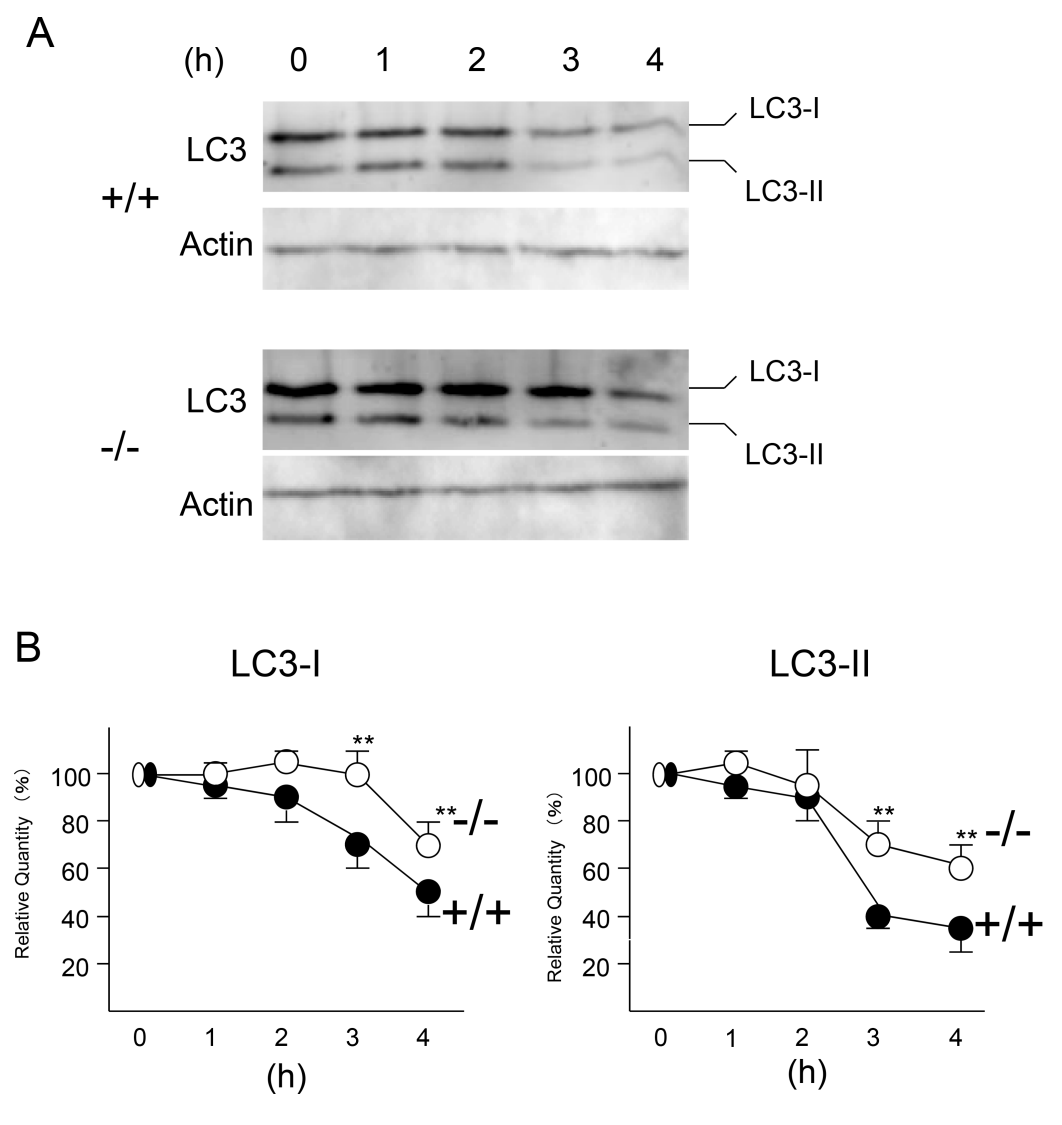
Changes of LC3 in wild-type and *CatE* ^*−/−*^ macrophages under starvation conditions. (**A**) Cultured macrophages derived from wild-type (+/+) and *CatE* ^*−/−*^ macrophages (−/−) were incubated with Hank’s balanced salt solution for the indicated times (0, 1, 2, 3, and 4). The cell lysates (100 µg protein for each) were subjected to SDS-PAGE followed by western blotting with specific antibodies to LC3. (**B**) Densitometric analysis for the quantification of each protein in the cell lysate of both cell types. The data was defined as the relative chemiluminescence intensity per mm^2^ measured by LAS1000. The data are indicated as the mean + SD values from 3 independent experiments. *P < 0.05 for the indicated comparisons.

Impaired fusion of the autophagosomes with lysosomes was also observed under starvation-induced conditions (Figure 4). When wild-type and *CatE* ^*−/−*^ macrophages were incubated with a nutrition-depleted medium, LC3-I and LC3-II of *CatE* ^*−/−*^ macrophages apparently remained, while those of wild-type macrophages gradually disappeared (Figure 4A). After 4 h of incubation, approximately 70 and 60 % of LC3-I and LC3-II, respectively, of *CatE* ^*−/−*^ macrophages were retained, whereas those of wild-type macrophages decreased to approximately 50% and 35%, respectively (Figure 4B). The results indicate that the degradation of LC3 in *CatE* ^*−/−*^ macrophages by starvation-induced autophagy was also impaired.

### Cathepsin E deficiency causes mitochondrial abnormalities in macrophages

**Figure 5:**
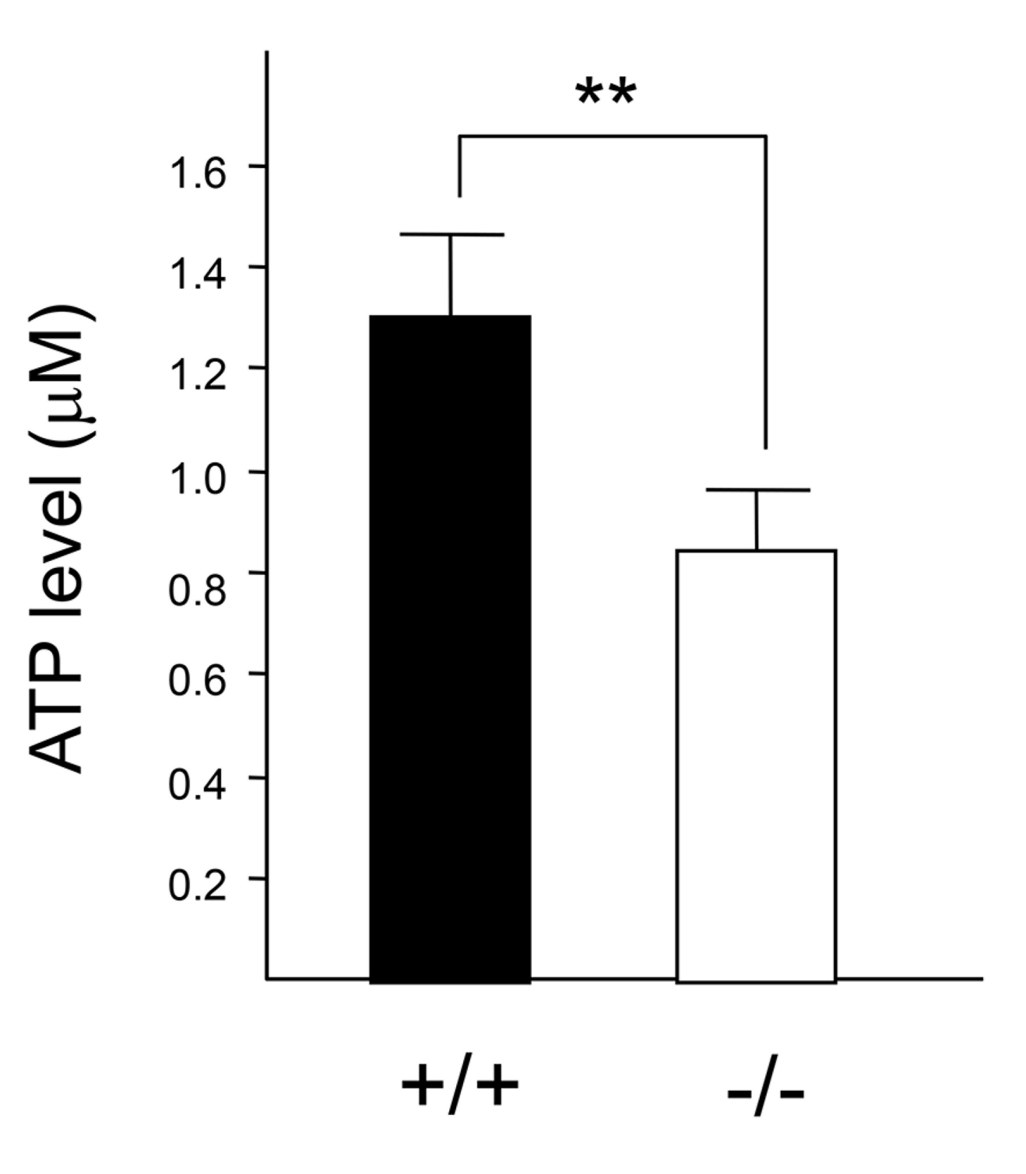
Intracellular ATP levels in wild-type and *CatE* ^*−/−*^ macrophages. Macrophages (1 × 10^5^ cells) were cultured on 96-well plates at 37°C for 24 h. After replacement with fresh media, the culture plate was incubated at room temperature (25°C) for 30 min. ATP levels were determined by the manufacturer’s protocol of the CellTiter-Glo assay kit according to the manufacturer’s protocol. The concentration of intracellular ATP was determined by the titration of the control medium without cells plus 0.1−1.0 µM ATP.

Autophagy is thought to regulate the turn-over of long-lived proteins and organelles in cells [13]. Given that cathepsin E deficiency inhibits autophagy, degradation of mitochondria may be delayed in *CatE* ^*−/−*^cells. To test the accumulation of abnormal mitochondria in *CatE* ^*−/−*^ macrophages, we measured ATP levels in both cell types. The ATP level in *CatE* ^*−/−*^ macrophages was significantly lower than that in wild-type cells (Figure 5). 

**Figure 6:**
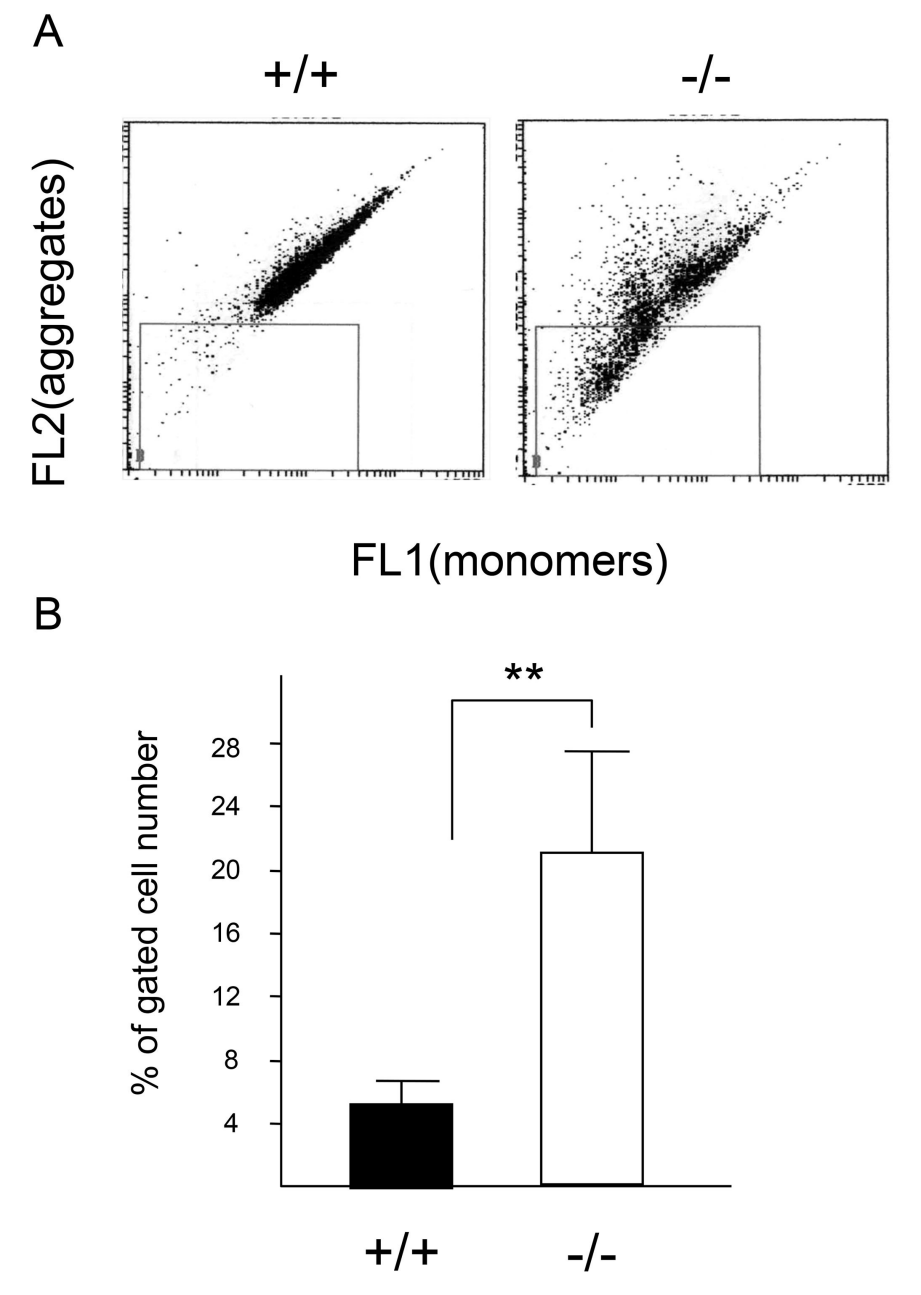
Mitochondrial membrane potential of wild-type and *CatE* ^*−/−*^ macrophages. (**A**). Mitochondrial membrane potential was measured with JC-1. A suspension of peritoneal macrophages (2 × 10^5^ cells/100 μL) was incubated on ice for 15 min with JC-1 in PBS containing 2.5% FBS and 0.01% NaN_3_. After washing, flow cytometric analyses were performed. (**B**) Comparison of the percentage of gated number of macrophages. Ten thousand cells were analyzed in each sample. The data are indicated as mean ± SD values of data from 4 independent experiments. *P < 0.05 for the indicated comparisons.

We further measured mitochondrial membrane potential with the MitoSensor JC-1, by flow cytometry. JC-1 is a cationic dye, which accumulates in the mitochondria in a potential-dependent manner, and the ratio of green to red fluorescence depends on the membrane potential only [31]. The JC-1 staining pattern of wild-type macrophages exhibited reddish fluorescence similar to that observed in normal mitochondria (Figure 6A). However, the staining pattern of mitochondria in *CatE* ^*−/−*^ macrophages changed to that of aggregates, indicating depolarization of the mitochondria (Figure 6A). The percentage of depolarized cells in CatE^−/−^ macrophages was significantly increased to approximately 22%, whereas that in wild-type macrophages was approximately 5% (Figure 6B).

**Figure 7:**
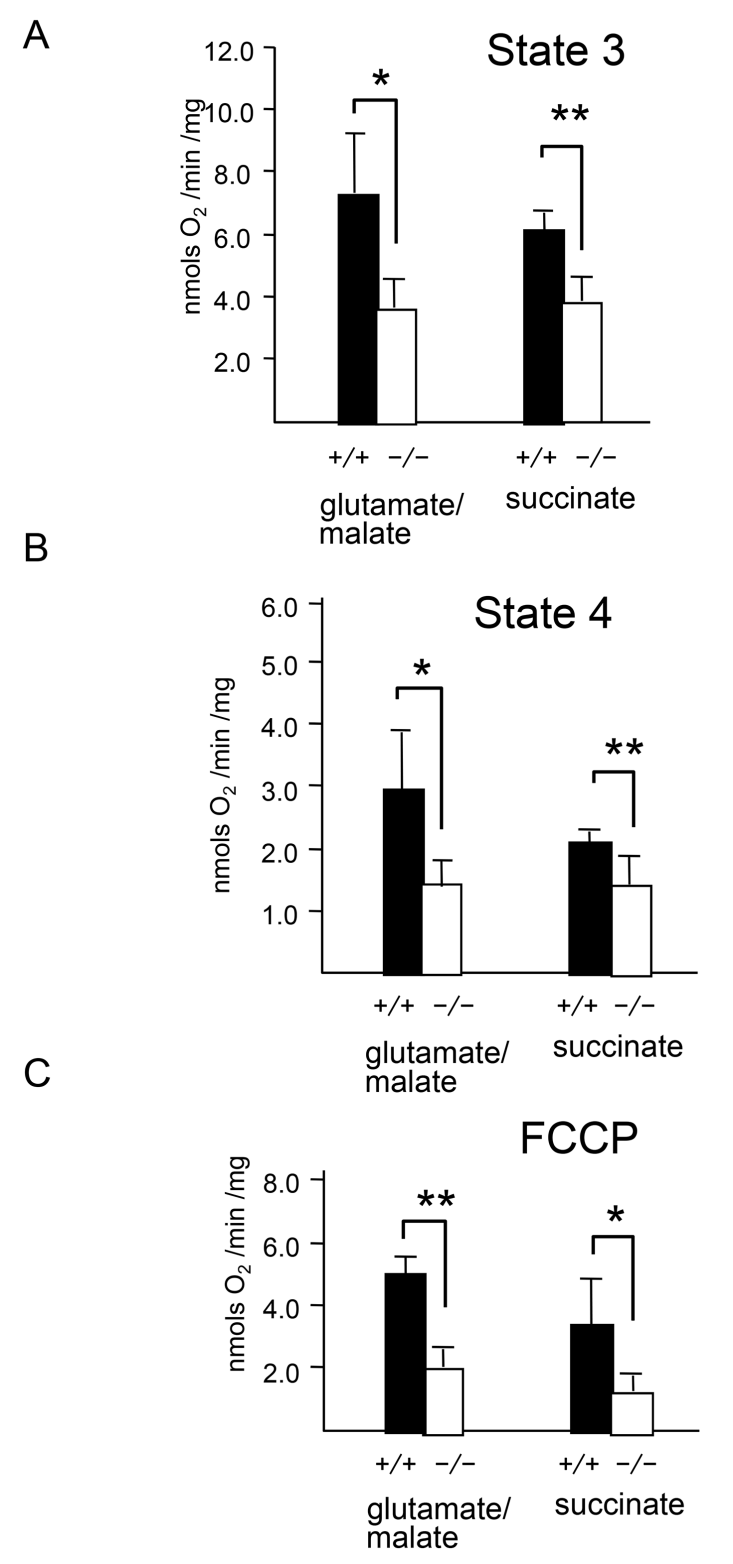
Mitochondrial respiration of wild-type and *CatE* ^*−/−*^ macrophages. After homogenization, mitochondrial fractions were isolated from macrophages. Respiratory substrate (10 mM glutamate plus 10 mM malate or 5 mM succinate with 10 µM rotenone) was then added and state 4 respiration was measured for 2 min; ADP (1.5 mM) was added, and state 3 respiration was measured for a further 2–4 min. The respiration rates were measured by an oxygraph. The rates were normalized to citrate synthase activity in the same samples. (**A**) state 3 (in the presence of ADP), (**B**) state 4 (in the absence of ADP) (**C**) the uncoupling agent FCCP.

Mitochondrial respiration is the most reliable parameter for measuring mitochondrial bioenergetics. Therefore, we measured the oxygen consumption of mitochondrial fractions from wild-type and *CatE* ^*−/−*^ macrophages by using substrates for mitochondrial complex I (glutamate/malate) or complex II (succinate) (Figure 7). In the presence (state 3) or absence of ADP (state 4), the oxygen consumption of the mitochondria isolated from *CatE* ^*−/−*^ macrophages was lower than that of wild-type cells (Figure 7A and B). Reduced respiration in mitochondria from *CatE* ^*−/−*^ macrophages was observed in the presence of carbonyl cyanide-p-trifluoromethoxyphenylhydrazone (FCCP), a protonophore used for mitochondrial respiration (Figure 7C). Thus, mitochondrial dysfunction was observed in *CatE* ^*−/−*^ macrophages. However, electron microscopic analysis did not reveal any morphological differences between wild-type and *CatE* ^*−/−*^ macrophages (data not shown).

### *CatE* ^*−/−*^ macrophages show an increased oxidative stress

**Figure 8:**
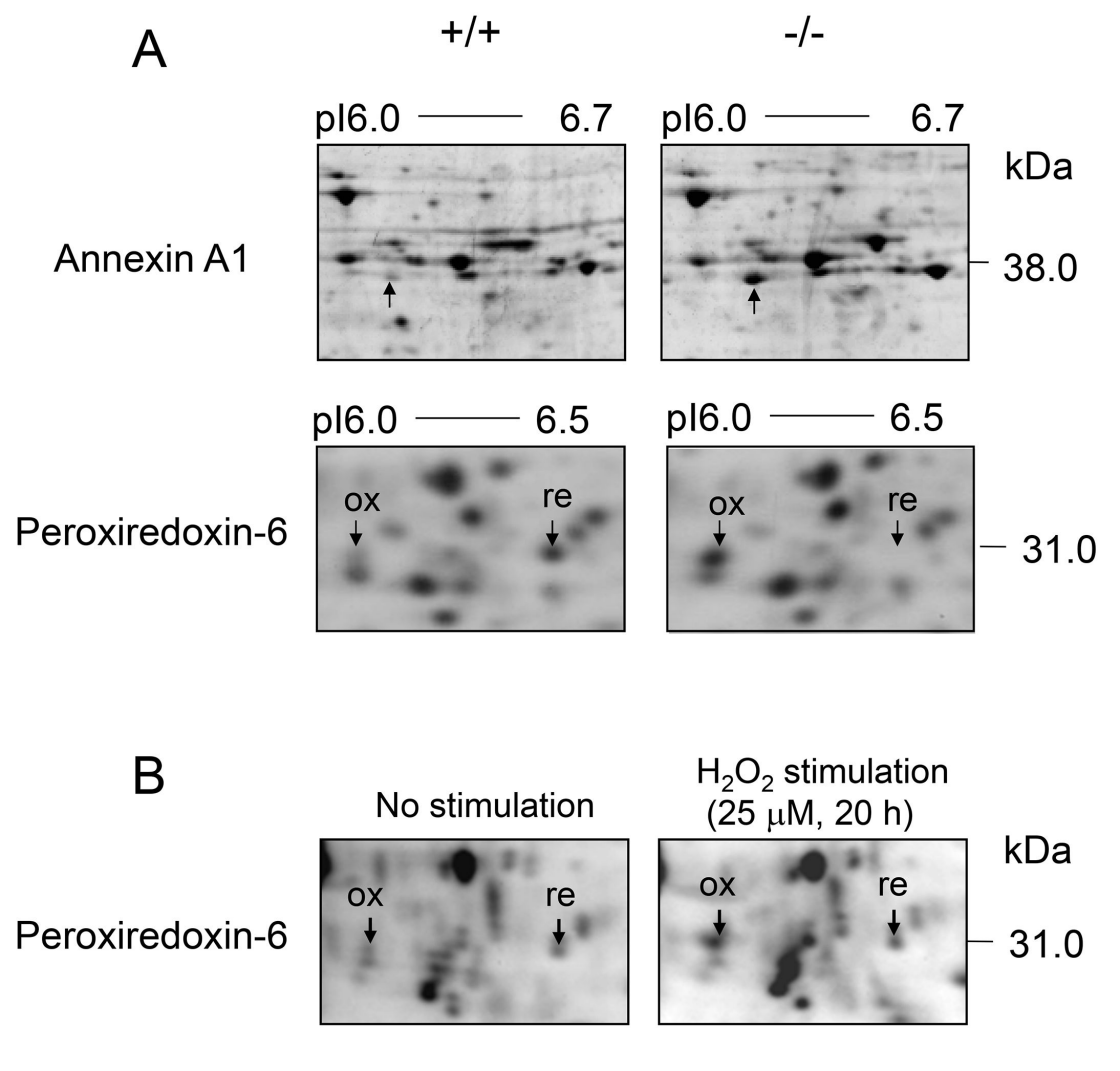
Two-dimensional gel maps of the cell lysates of macrophages. (**A**) Proteome analysis of wild-type and *CatE* ^*−/−*^ macrophages revealed that almost all the spots located in the areas under pI 6.0–6.7 were identified annexin A_1_, and those under pI 6.0–6.5 were identified the oxidized and reduced forms of peroxiredoxin-6 respectively. These data indicate the representative 2-D gel maps of 3 independent experiments. (**B**) Proteomic analysis with wild-type macrophages treated with H_2_O_2_ or untreated.

Next, we hypothesized that the abnormal mitochondria in CatE^−/−^ macrophages might affect molecules other than mitochondria, such as cytosolic proteins. To identify proteins that had been changed by cathepsin E deficiency, we examined high resolution 2-D gel electrophoresis within a pI range of 4−7 and a molecular mass range of 15−200 kDa, and compared the proteomic profiles of the cell lysates from both wild-type and *CatE* ^*−/−*^ macrophages. The 2-D gel electrophoresis analysis detected more than 100 spots. After comparison of the spot volumes, we identified 3 series of protein spots having apparent molecular masses of 38 kDa (pI 6.0−6.7) (a) and 31 kDa (pI 6.0−6.5) (b) that were different in CatE^−/−^ macrophages in comparison to wild-type cells (Figure 8). Proteomic analysis showed that the 38 kDa spot that increased in CatE^−/−^ macrophages was identical with annexin A_1_. It is known that annexin A_1_ is a multifunctional protein that is up-regulated by inflammatory and oxidative stimulation (33). In contrast, the two spots of approximately 31 kDa, which increased and decreased, were identified as peroxiredoxin-6. Peroxiredoxin-6 is an antioxidative protein existing in two forms: A reduced form and an oxidized form [32] [33]. To confirm the intracellular conformation of peroxiredoxin-6, we treated wild-type macrophages with hydrogen peroxide (H_2_O_2_) analyzed by the 2-D gel electrophoretic profile. Upon H_2_O_2_ stimulation, the acidic spot of peroxiredoxin-6 showed an apparent increase, whereas the basic spot a slight decrease (Figure 8B). In addition to these results, various studies have suggested that impairments in the electron transport system in mitochondria are associated with decreased energy production and increased formation of reactive oxygen species (ROS) [34] [35]. Taken together, these results with increased annexin A_1_ and oxidized peroxiredoxin-6 suggest increased oxidative stress in *CatE* ^*−/−*^ macrophages.

**Figure 9:**
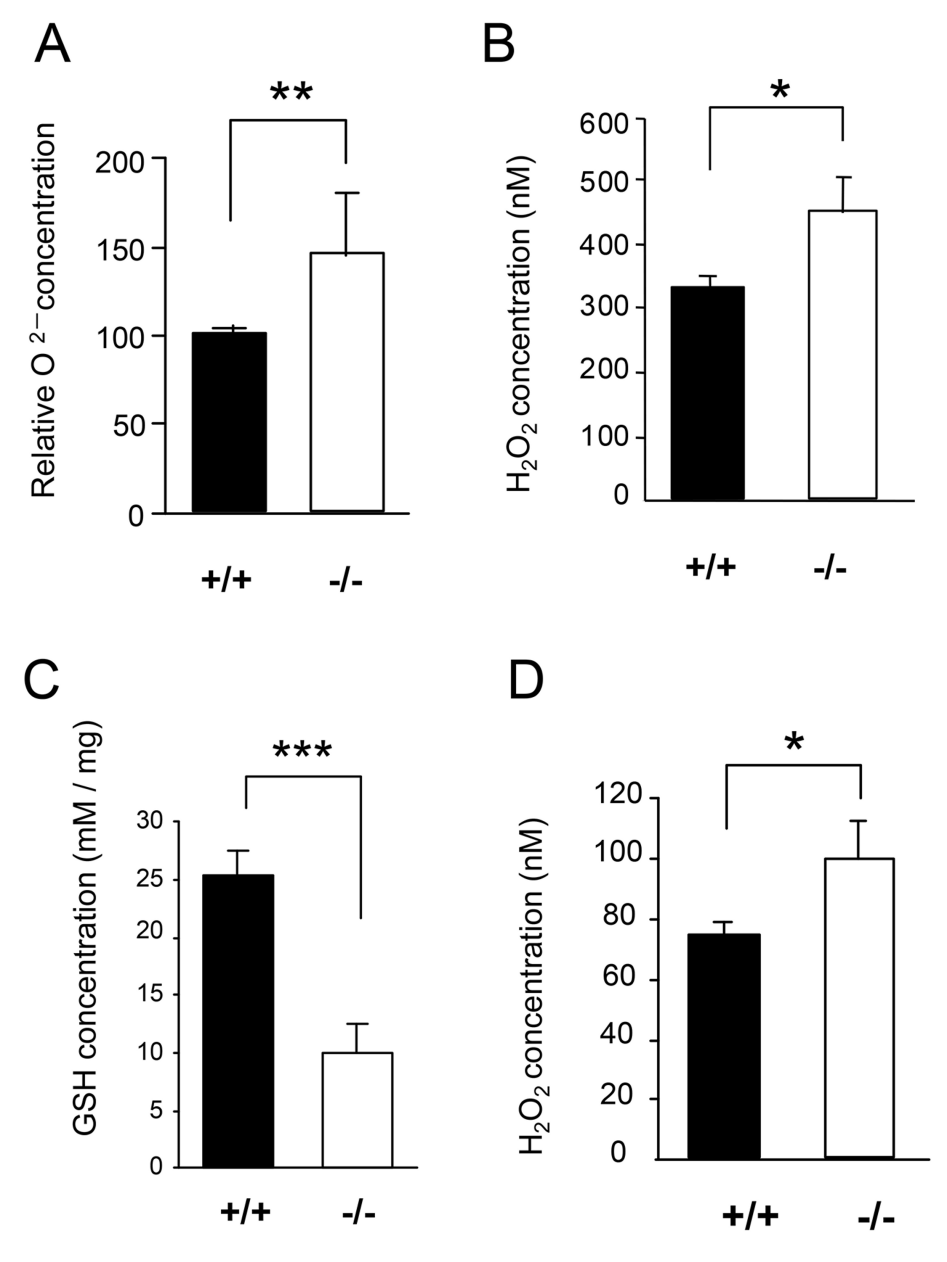
Levels of oxidative burst, H_2_O_2_ production, and reduced glutathione in wild-type and *CatE* ^*−/−*^ macrophages. (**A**) Levels of oxidative burst by macrophages. Macrophages in cell suspension (1 × 10^7^ cells/mL) were incubated with zymosan suspension at 37 °C for 30 min. Levels of oxidative burst of macrophages were determined by a luminophotometer. (**B**) The levels of H_2_O_2_ production of macrophages. Macrophages (1 × 10^6^ cells/50 μL) were cultured without any stimulation at 37°C for 1 h. Determination of H_2_O_2_ production into the culture media by macrophages was performed by an Amplex Redassay kit. (**C**) The GSH levels of cell lysates of macrophages were assayed with NADPH, measuring the development with DTNB at 412 nm with spectrophotometer. (D) Levels of H_2_O_2_ in the serum (50 μL) of mice were determined by an Amplex Redassay kit. The data are the means ± SD of values from 4 independent experiments. *P <0.05, **P <0.01, ***P <0.001.

To further test the increased oxidative stress in *CatE* ^*−/−*^ macrophages, we measured oxidative burst in macrophages by chemiluminescence after stimulation with zymosan (Figure 9A). The relative concentration of superoxide (O_2_^−^) in *CatE* ^*−/−*^ macrophages was approximately 150% that of wild-type cells (Figure 9A). However, the induced oxidative burst was thought to result from a mixture of O_2_^−^ from NADPH oxidase in phagosomes and endogenous O_2_^−^ from mitochondrial respiration. Therefore, to exclude ROS production derived from NADPH oxidase, we measured the levels of H_2_O_2_ in the extracellular media of macrophages without any stimulation. As shown in Figure 8B, the H_2_O_2_ levels were significantly higher in media from *CatE* ^*−/−*^ macrophages than in those from wild-type cells. We also measured the intracellular levels of glutathione (GSH), which is an antioxidant tripeptide and a useful marker of the redox state [36]. The levels of GSH were markedly diminished in *CatE* ^*−/−*^ macrophages compared with wild-type cells (Figure 9C). Thus, *CatE* ^*−/−*^ macrophages apparently exhibited increased oxidative stress in terms of increased H_2_O_2_ and decreased GHS levels. Importantly, we found that H_2_O_2_ levels in serua from *CatE* ^*−/−*^ mice were also significantly increased compared with those in wild-type mice (Figure 9D). These results indicate that oxidative stress was increased in *CatE* ^*−/−*^ cells through an NADPH oxidase-independent pathway including mitochondrial respiration.

## Discussion

In this study, we demonstrated that cathepsin E deficiency resulted in increased levels of the autophagy marker proteins LC3, p62, and polyubiquitinated proteins. In addition, cathepsin E deficiency caused delayed turnover of long-lived proteins, and impaired fusion of autophagosome to lysosomes in *CatE* ^*−/−*^ macrophages. These results indicate that cathepsin E deficiency causes impairment of autophagy. However, *CatE* ^*−/−*^ macrophages display mitochondrial abnormalities such as decreased intracellular ATP levels, depolarized mitochondrial membrane potential, and decreased mitochondrial oxygen consumption. Although 3-MA and Baf have effects on autophagy-lysosome, but these inhibitors are thought to have little, if any, direct inhibitory effect on mitochondrial function. Taken together, we conclude that in macrophages, cathepsin E is essential for eliminating abnormal mitochondria by the autophagy process.

In *CatE* ^*−/−*^ macrophages, the impaired autophagic flux is probably a secondary event. Our previous study demonstrated that *CatE* ^*−/−*^ macrophages displayed accumulation of major lysosomal membrane glycoproteins, and followed by elevated lysosomal pH and enhanced soluble lysosomal enzyme secretion [10]. These primary events, including accumulating LAMPs and elevated lysosomal pH presumably causes secondary events related to abnormal membrane trafficking. In fact, *CatE* ^*−/−*^ macrophages show decreased surface levels of various receptors [9] [12]. Similarly, the accumulation of LC3 proteins and impaired fusion of autophagosomes to lysosomes also result in abnormal membrane trafficking in *CatE* ^*−/−*^ macrophages. The amount of the membrane form of LC3-II correlates with the number of autophagosomes [24]; hence, the findings that *CatE* ^*−/−*^ macrophages accumulate both the cytoplasmic LC3-I and the membrane-bound LC3-II imply that both membrane trafficking and degradation are impaired.

The phenotype of *CatE* ^*−/−*^ macrophages with impaired autophagy is similar to that of cells lacking lysosomal proteins, although cathepsin E is an endosomal proteinase. For example, LC3 protein levels are elevated in neuronal ceroid lipofuscinosis (Batten diseases) [37], multiple sulfate deficiency, mucopolysaccharidosis type IIIA [30], and Niemann-Pick Type C disease [38]. Moreover, accumulation of p62 and polyubiquitinated proteins has been reported in murine cells deficient in autophagy (*Atg*-*5* or *Atg*-7 deficient mice) [27,28]. Because p62 directly associates with LC3 to promote the degradation of poly-ubiquitinated protein aggregates by autophagy [31], the intracellular p62 accumulation also indicates impaired autophagy. Thus, our findings indicate that the endosomal protease cathepsin E is involved in autophagy-lysosome degradation systems in macrophages.

The impaired autophagic flux in *CatE* ^*−/−*^ macrophages resulted in various mitochondrial abnormalities. These results presumably imply an accumulation of abnormal mitochondria in *CatE* ^*−/−*^ macrophages due to decreased mitochondrial degradation and thereby impaired membrane trafficking. This notion is supported by data showing decreased fusion of autophagosomes to lysosomes in *CatE* ^*−/−*^ macrophages. Similar results were observed in mice deficient in other cathepsins. Koike et al. demonstrated that cathepsin D single-deficient or cathepsins B and L double-deficient mice show the accumulation of LC3 and autophagosomes in the brain [37]. We therefore speculate that the lack of morphological abnormalities in *CatE* ^*−/−*^ macrophages were due to the amounts of lysosomal proteinases. It is known that professional phagocytic macrophages contain large amounts of lysosomal proteinases [39,40], whereas neurons contain small amounts of lysosomal proteinases. Therefore, it would be difficult to detect the abnormal accumulation of autophagic substrates, including abnormal mitochondria, in macrophages.

Autophagy-related signaling was perturbed in *CatE* ^*−/−*^ macrophages. Autophagy is regulated by two major signaling pathways in response to starvation: the class I phosphatidylinositol 3-phosphate kinase/Akt/mTOR/S6K/AMPK signaling pathway and the Ras/Raf-1/ ERK/ JNK pathway [41] [42]. The former pathway negatively regulates autophagy, while the latter pathway positively regulates autophagy [41] [42]. In case of *CatE* ^*−/−*^ macrophages, however, both the mTOR/Akt/AMPK and the ERK pathways were activated, whereas S6K was inactivated. Therefore, the perturbed signaling pathways are likely to correlate with increased oxidative stress in *CatE* ^*−/−*^ macrophages. In fact, a recent study has reported that there is a relationship between the up-regulation of Akt/mTOR and ERK signaling pathways and the increased ROS activity in other lysosomal storage disorders [43]. Since major intracellular sources of ROS are thought to be derived from dysfunctional mitochondria with impaired respiration [44], the increased ROS production in *CatE* ^*−/−*^ macrophages is probably due to the accumulation of dysfunctional mitochondria resulting from impaired autophagy.

## Conclusion

*CatE* ^*−/−*^ macrophages display inhibited autophagy, which results in increased numbers of abnormal mitochondria and ROS production, implying that cathepsin E is an essential proteinase for mitophagy in macrophages.
