# Correction: Cathepsin E Deficiency Impairs Autophagic Proteolysis in Macrophages

**DOI:** 10.1371/journal.pone.0099278

**Published:** 2014-05-27

**Authors:** The PLOS ONE Staff

Figure 2 and its figure legend are incorrect. The authors have provided a corrected version here.

**Figure 2:**
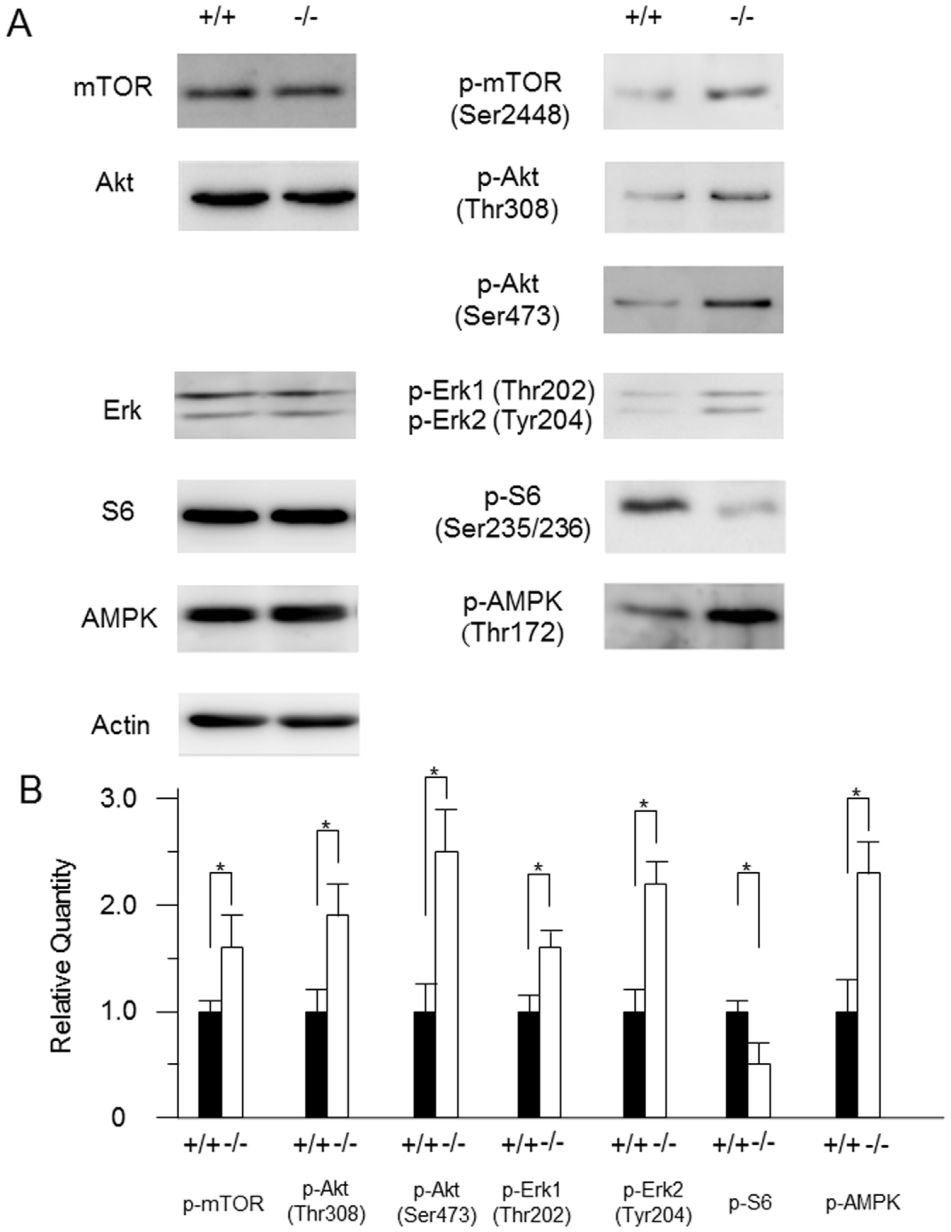
Comparison of Akt/mTOR and ERK signaling pathways in wild-type and *CatE^−/−^*  macrophages. (**A**) The cell lysates (100 µg protein for each) derived from wild-type (+/+) and *CatE* ^−/−^ macrophages (−/−) were subjected to SDS-PAGE followed by western blotting with specific antibodies to mTOR, p-mTOR, Akt, p-Akt(Thr308), p-Akt(Ser473), Erk, p-Erk1/2(Thr202/Tyr204), S-6, p-S6, AMPK, p-AMPK and actin. The data indicate the representative Western blotting of 3 independent experiments. (**B**) Densitometric analysis for the quantification of each protein in the cell lysate of both cell types. The arbitrary density unit was defined as the relative chemiluminescence intensity per mm^2^ measured by LAS1000. The data are indicated as the mean ± SD values from 3 independent experiments. *P < 0.05 for the indicated comparisons.
